# Multifunctional biocompatible chitosan-polypyrrole nanocomposites as novel agents for photoacoustic imaging-guided photothermal ablation of cancer

**DOI:** 10.1038/srep43593

**Published:** 2017-03-02

**Authors:** Panchanathan Manivasagan, Nhat Quang Bui, Subramaniyan Bharathiraja, Madhappan Santha Moorthy, Yun-Ok Oh, Kyeongeun Song, Hansu Seo, Min Yoon, Junghwan Oh

**Affiliations:** 1 Marine-Integrated Bionics Research Center, Pukyong National University, Busan 48513, Republic of Korea; 2 Department of Biomedical Engineering and Center for Marine-Integrated Biotechnology (BK21 Plus), Pukyong National University, Busan 48513, Republic of Korea; 3 Department of statistics, College of Natural of Sciences, Pukyong National University, Busan, 48513, Republic of Korea

## Abstract

Cancer nanotechnology is emerging as one of the promising strategies combining photothermal therapy (PTT) and photoacoustic imaging (PAI) for the treatment of breast cancer and it has received considerable attention in the recent years because it is minimally invasive, prevents damage to non-targeted regions, permits fast recovery, and involves breast cancer imaging. The present study demonstrates multifunctional biocompatible chitosan-polypyrrole nanocomposites (CS-PPy NCs) as novel agents for photoacoustic imaging-guided photothermal ablation of cancer because of their biocompatibility, conductivity, stability, and strong near-infrared (NIR) absorbance. The CS-PPy NCs are spherical in shape and range 26–94 nm in size with a mean value of 50.54 ± 2.56 nm. The *in vitro* results demonstrated good biocompatibility of CS-PPy NCs, which can be used in PTT for cancer cells under 808-nm NIR laser irradiation. Tumor-bearing mice fully recovered after treatment with CS-PPy NCs and NIR 808-nm laser irradiation compared to the corresponding control groups. Our research highlights the promising potential of using CS-PPy NCs for photoacoustic imaging-guided photothermal ablation of cancer in preclinical animals, which should be verified in future clinical trials.

Development of new nanomaterials for breast cancer theranostics that combines therapeutic and diagnostic applications has attracted much attention in the recent decades^1^,^2^,^3^,^4^. For efficient breast cancer treatment, development of novel nanomaterials capable of reacting to the local tumor environment is required^5^,^6^,^7^. Light-activated theranostics has received considerable attention as one of the major research areas in the recent decades, particularly in photothermal therapy (PTT)^8^. PTT has become popular in modern cancer therapy because it successfully kills cancer cells in a target area through heat induced hyperthermia, minimally invasive, permits fast recovery, and prevents damage to non-targeted regions^9^,^10^,^11^,^12^. Compared to the traditional approaches including surgery, chemotherapy, and radiotherapy, PPT is more efficient in tumor ablation and involves minimal risk of damage to healthy tissues^13^. In recent years, many researchers have focused on developing light-absorbing nanoparticles as novel photothermal agents with strong absorption and scattering in the NIR region, which is a biological transparency^14^,^15^,^16^,^17^. The ability to produce hyperthermic temperatures (>43 °C) at a specific area with externally tunable controls has a major advantage over inducing full-body hyperthermia for cancer therapy^18^.

Human breast cancer is the second most common cause of cancer-related deaths in women and the incidence of breast cancer has increased worldwide in the last few years^19^. Chemotherapy plays a major role in the management and treatment of cancer. The major drawbacks of chemotherapy are its whole body effects, high price, and low level of efficiency^20^. Therefore, there is an urgency to develop efficient methods that minimize the side effects of chemotherapy by specifically delivering the chemotherapeutic agent to only the tumor cells^21^. PTT is rapidly emerging as one of the promising methods for cancer therapy. Photoacoustic imaging (PAI) is a recently developed technique of biomedical imaging that combines the advantages of optical and acoustic imaging^22^,^23^,^24^. PAI has been applied in the various medical fields for obtaining anatomic and functional information, breast cancer imaging, brain structural and functional imaging, and quantitative blood flow estimation^25^,^26^,^27^. Therefore, it is undoubted that the PAI would conduce to accurate locating of cancerous tissue for more precise guidance for PTT.

Recently, several nanoparticles (gold nanoparticles (AuNPs), copper sulfide nanoparticles (CuS NPs), polypyrrole nanoparticles (PPy NPs), carbon nanomaterials, prussian blue nanoparticles (PB NPs), and upconversion nanoparticles (UCNPs)) with strong NIR absorbance have been investigated for photothermal therapy^28^. Because of morphology-dependent NIR absorbing property, gold nanorods, gold nanocages, and gold nanoshells have been explored as photoablation agents for cancer therapy^18^. However, these NIR absorbing AuNPs have low photostability since their morphology and NIR absorption peak would diminish after a long period of laser irradiation owing to the “melting effect”^29^. Among all the NIR absorbing nanoparticles, CS-PPy NCs are of particular importance as photoablation agents for localized tumorous PTT because of the excellent biocompatibility, significant photothermal conversion efficacy, and remarkable photostability higher than AuNPs.

Marine biopolymers are attractive biomaterials for clinical applications owing to their excellent biocompatibility, biodegradability, inexpensiveness, abundance, stability, ease of surface modification, and nontoxic nature^30^,^31^,^32^. Chitosan (CS) is a natural biopolymer obtained by the *N*-deacetylation of chitin. CS has received considerable attention in the recent years for its applications in the cosmeceutical, nutraceutical, and pharmaceuticals industries^33^. Polypyrrole (PPy) is an organic conductive polymer and it is widely used in organic electronics because of its excellent conductivity, stability, and strong NIR absorbance^34^. PPy is highly promising in various biomedical applications^35^. Combining the characteristics of these materials as chitosan-polypyrrole nanocomposites (CS-PPy NCs) could potentially enhance their biocompatibility, stability, conductivity, and strong NIR absorbance. In the present study, we report the CS-PPy NCs for the photoacoustic imaging guided photothermal ablation of cancer. The CS-PPy NCs were characterized *via* various methods. The photothermal effect performance of the CS-PPy NCs was proven *in vitro* and *in vivo* under 808-nm NIR laser irradiation. Additionally, their efficiency in PAI was proven *in vivo* by scanning mice intratumorally injected with CS-PPy NCs.

## Results and Discussion

**Figure 1:**
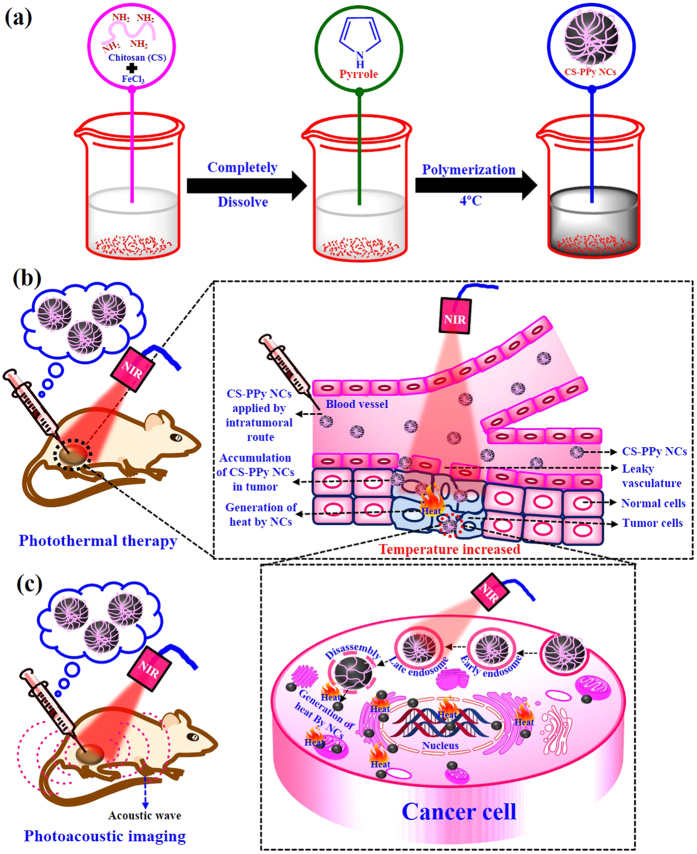
(**a**) Schematic for the preparation of chitosan-polypyrrole nanocomposites (CS-PPy NCs). A scheme showing the possible mechanism of CS-PPy NCs for photothermal therapy (**b**) and photoacoustic imaging (**c**) of cancer.

**Figure 2:**
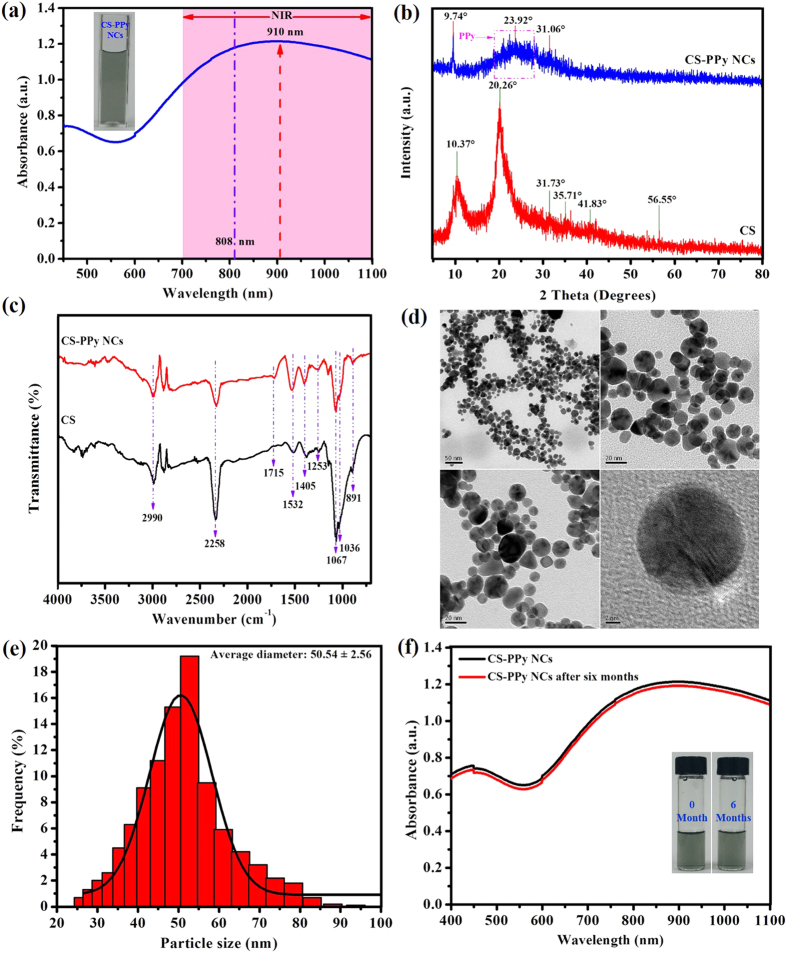
(**a**) UV-Vis-NIR absorbance spectrum recorded for 150 μg/mL dispersion of CS-PPy NCs in water (The inset shows CS-PPy NCs solution). (**b**) XRD patterns of chitosan (CS) and CS-PPy NCs. (**c**) FTIR spectrum of CS and CS-PPy NCs. (**d**) FETEM image of CS-PPy NCs. (**e**) Particle size distribution histogram obtained from DLS fitted to the log-normal distribution curve. (**f**) UV-Vis-NIR absorbance spectrum recorded for 150 μg/mL dispersion of CS-PPy NCs in water and stored at room temperature for 6 months (The inset shows the freshly prepared CS-PPy NCs solution and the 6-month old CS-PPy NCs solution).

Figure 1 shows the fabrication of CS-PPy NCs (Fig. 1a) for photoacoustic imaging-guided photothermal ablation of cancer (Fig. 1b and c). The formation of CS-PPy NCs was indicated by a visual color change in the reaction solutions. A black color was quickly formed within 3 min, indicating the synthesis of CS-PPy NCs, and the reaction solutions were observed using UV-Vis spectroscopy. The maximum peak was observed at 910 nm, which confirmed strong absorption and scattering in the NIR region (700–1100 nm) owing to the localized surface plasmon resonance (SPR) (Fig. 2a). In addition, CS-PPy NCs have been used for photothermal ablation and photoacoustic imaging. Figure 2b shows the XRD patterns of the CS-PPy NCs as well as those of the parent CS and PPy. The XRD pattern of CS shows major characteristic peaks at 10.37°, 20.26°, and 31.73°. A broad peak at 23.92° indicates that the PPy is in the amorphous form. The XRD pattern of CS-PPy NCs displays three characteristic peaks at 9.74°, 23.92°, and 31.06°, which clearly displays the characteristic peaks associated with CS and PPy.

The composition of the CS and CS-PPy NCs were further characterized by FTIR spectroscopy (Fig. 2c). The FTIR spectra of CS and CS-PPy NCs showed bands at 891 cm^−1^, 1036 cm^−1^, 1067 cm^−1^, 1253 cm^−1^, 1405 cm^−1^, 1532 cm^−1^, 1715 cm^−1^, 2258 cm^−1^, and 2990 cm^−1^. A characteristic strong amine (N–H) peak was noticed at 891 cm^−1^. The peaks at 1036 cm^−1^ and 1067 cm^−1^ correspond to the C–N stretching of aliphatic amines. The strong bands detected at 1253 cm^−1^ correspond to the C–N stretching of aromatic amines. A peak of considerable intensity at 405 cm^−1^ is assigned to the C–C stretching of aromatics. The strong bands observed at 1532 cm^−1^ were assigned to the N–O asymmetric stretching of nitro compounds. The characteristic peak at 1715 cm^−1^ (C = O) was observed with considerable intensity. The peak at 2258 cm^−1^ corresponds to the –C≡C– stretching of alkynes. The medium bands detected at 2990 cm^−1^ correspond to the C–H stretching of alkanes.

The morphology of the CS-PPy NCs was characterized by FETEM. The FETEM image in Fig. 2d shows that the CS-PPy NCs are spherical in shape and their sizes range from 26 to 94 nm (Fig. S1). The DLS histogram shows that the CS-PPy NCs are well dispersed with particle sizes ranging from 26 to 94 nm with a mean value of 50.54 ± 2.56 nm (Fig. 2e). The ZP of the CS-PPy NCs solution was determined to be +58.36 mV, indicating positive surface charge and stability because colloids with ZPs higher than 30 mV (positive or negative) are commonly stable. The stability of CS-PPy NCs is one of the most important parameters for biomedical applications. Therefore, the stabilities of freshly prepared and 6-month old CS-PPy NCs were compared (Fig. 2f). There was no significant difference between the UV-Vis spectra of the freshly prepared CS-PPy NCs solution and the 6-month old solution, which suggested the good colloidal stability.

### Photothermal heating performance

**Figure 3:**
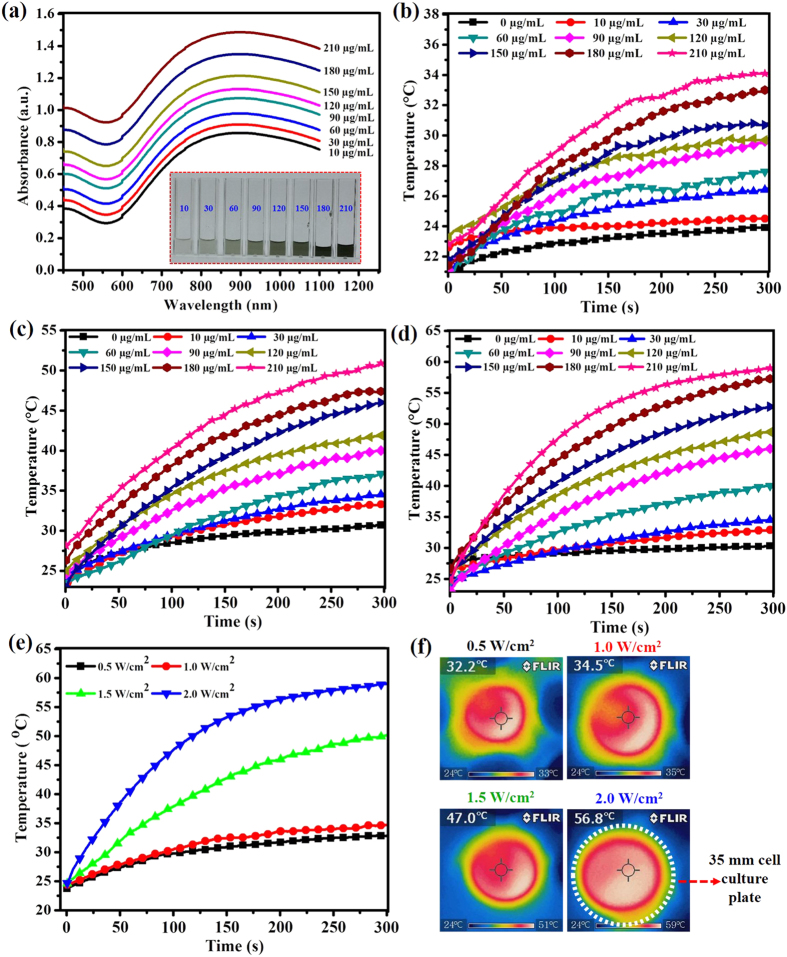
(**a**) UV-Vis-NIR absorbance spectrum of different concentrations of CS-PPy NCs in water (The inset photograph shows different concentrations of the CS-PPy NCs solution). The temperature elevation in an aqueous solution of different CS-PPy NCs concentrations (0, 10, 30, 60, 90, 120, 150, 180, and 210 μg/mL) as a function of irradiation time by 808 nm laser at different laser power densities: 1.0 W/cm^2^ (**b**), 1.5 W/cm^2^ (**c**), and 2.0 W/cm^2^ (**d**). (**e**) The temperature elevation of CS-PPy NCs aqueous solution at a concentration of 210 μg/mL under 808-nm laser irradiation at different power densities (0.5 W/cm^2^, 1.0 W/cm^2^, 1.5 W/cm^2^, and 2.0 W/cm^2^) for 5 min. (**f**) NIR thermographic images of CS-PPy NCs (210 μg/mL) aqueous solution under exposure to an 808-nm NIR laser at different power densities (0.5 W/cm^2^, 1.0 W/cm^2^, 1.5 W/cm^2^, and 2.0 W/cm^2^) for 5 min.

Different concentrations of CS-PPy NCs (10, 30, 60, 90, 120, 150, 180, and 210 μg/mL) were dispersed in distilled water and they were observed by UV-vis spectroscopy (Fig. 3a and Fig. S2). Different CS-PPy NC concentrations were scanned in the range from 400 to 1100 nm. Strong NIR absorption, water solubility, biocompatibility, and conductivity make CS-PPy NCs potential novel photothermal agents. Different concentrations of CS-PPy NCs were exposed to 808-nm NIR laser irradiation at different power densities (0.5, 1.0, 1.5, and 2.0 W/cm^2^). The photothermal heating effect of CS-PPy NCs increased with concentration and NIR laser power density, which suggested that temperature elevation was dependent upon CS-PPy NCs concentration and NIR laser power density (Fig. 3b–d and Fig. S3a–c). It was observed that a 5 min light exposure (2 W/cm^2^) resulted in a temperature elevation of 30.3, 32.8, 34.4, 40.0, 45.9, 48.6, 52.7, 57.3, and 58.9 °C at CS-PPy NCs concentrations of 0, 10, 30, 60, 90, 120, 150, 180, and 210 μg/mL, respectively (Fig. S4). The magnitude of temperature elevation increased with CS-PPy NCs concentration. In contrast, no noticeable temperature change was recorded when pure water was exposed to 808-nm NIR laser in the absence of CS-PPy NCs. CS-PPy NCs (210 μg/mL) were exposed to NIR laser irradiation at different laser power densities of 0.5, 1.0, 1.5, and 2.0 W/cm^2^ for 5 min, which resulted in a temperature elevation of 32.9, 34.6, 49.9, and 58.9 °C, respectively (Fig. 3e and Fig. S5). Thermographic images in Fig. 3f also indicated that CS-PPy NCs can efficiently induce thermal conversion when irradiated with a NIR laser. These results confirmed that CS-PPy NCs could act as effective photothermal agents for photoablation of tumors.

**Figure 4:**
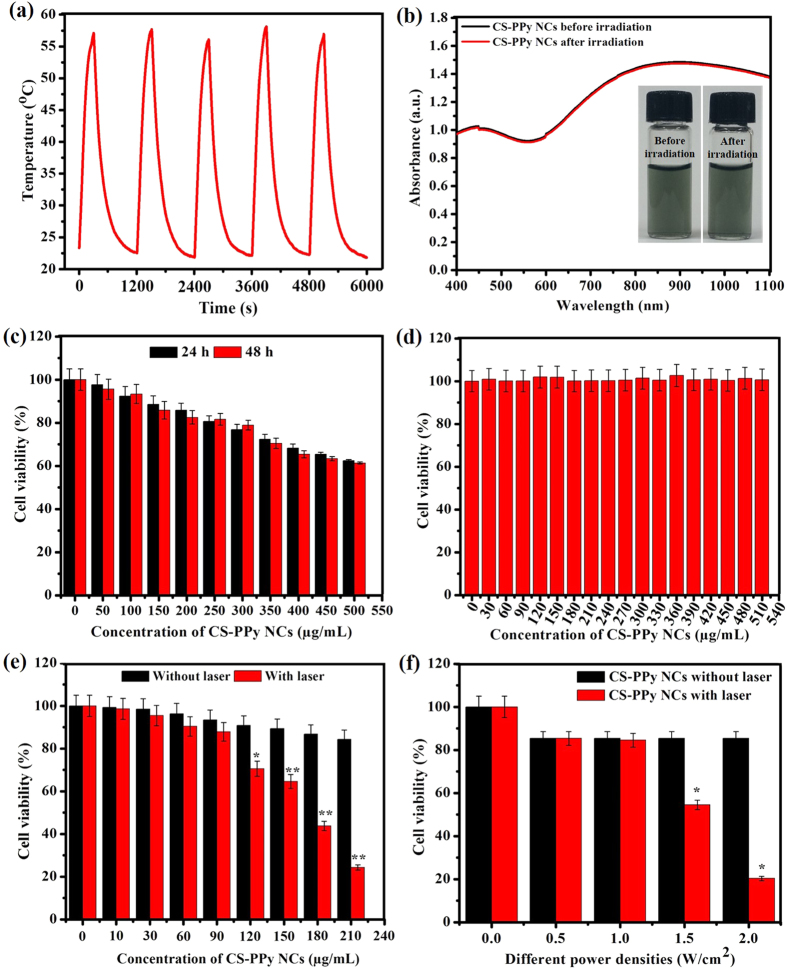
(**a**) Temperature elevation of CS-PPy NCs solution at the concentration of 210 μg/mL during for successive five cycles of an on/off NIR laser irradiation (2.0 W/cm^2^). (**b**) UV-Vis-NIR absorbance spectrum of CS-PPy NCs in water before and after five laser on/off of NIR light (The inset shows CS-PPy NCs solution in water before and after laser). (**c**) *In vitro* cytotoxic effect of CS-PPy NCs against MDA-MB-231 cells at 24 and 48 h. Data presented as mean ± standard deviation (n = 3). (**d**) Biocompatibility of CS-PPy NCs against HEK 293 cells for 24 h. Data presented as mean ± standard deviation (n = 3). (**e**) Cell viability assay results for MDA-MB-231 cells after exposure to different concentrations of CS-PPy NCs with or without 808-nm NIR laser irradiation at 2.0 W/cm^2^ for 5 min. Data presented as mean ± standard deviation (n = 3) (*Significant *p* < 0.05; **highly significant *p* < 0.01). (**f**) Cell viability of MDA-MB-231 cells after treatment with or without CS-PPy NCs (210 μg/mL) under irradiation of different laser power densities (0.5 W/cm^2^, 1.0 W/cm^2^, 1.5 W/cm^2^, and 2.0 W/cm^2^) for 5 min. Data presented as mean ± standard deviation (n = 3) (*Significant *p* < 0.05).

To further investigate the NIR photostability of CS-PPy NCs, five cycles of laser on/off irradiation were used and a CS-PPy NCs solution of 210 μg/mL was irradiated with 808-nm NIR laser at 2.0 W/cm^2^ for 5 min (laser ON), followed by natural cool down to room temperature without NIR laser irradiation for 30 min (laser OFF). As shown in Fig. 4a, after 5 cycles of on/off laser irradiation, no notable decrease in temperature elevation was observed during the experiment. Furthermore, the UV-Vis spectra of CS-PPy NCs showed no obvious decrease in the absorbance at 808-nm after five cycles of laser on/off (Fig. 4b). These results indicated that CS-PPy NCs showed excellent photostability after a long period of NIR laser irradiation, which could be beneficial for cancer therapy.

### *In vitro* cytotoxicity assay

A major concern about the biomedical application of nanomaterials is their toxicity. The cytotoxicity of CS-PPy NCs for MDA-MB-231 cells was examined using the standard MTT assay. As presented in Fig. 4c, although cell viability was slightly reduced in a dose-and time-dependent manner, no significant cytotoxicity was observed after 24 and 48 h incubation with any concentration of CS-PPy NCs. Even after 48 h exposure to the highest concentration of CS-PPy NCs (500 μg/mL), the viability of the cell population was more than 60%, indicating a very low cytotoxicity and good biocompatibility for the CS-PPy NCs. Biocompatibility of nanomaterials has been considered to be essential for their biomedical applications. The biocompatibility test was conducted for 24 h and 48 h on HEK 293 cells in the concentration range from 30 to 510 μg/mL (Fig. 4d and Fig. S6). Our results showed that CS-PPy NCs are non-toxic and have excellent biocompatibility, suggesting their suitability for *in vivo* studies.

### *In vitro* photothermal ablation of cancer cells

The *in vitro* photothermal effect of CS-PPy NCs was further investigated using a quantitative MTT assay. Quantitative analysis of photothermal effect was performed after 808-nm NIR irradiation (2 W/cm^2^) of MDA-MB-231 cells treated with difference concentrations of CS-PPy NCs (10, 30, 60, 90, 120, 150, 180, and 210 μg/mL) for 5 min. As shown in Fig. 4e, MDA-MB-231 cells treated with CS-PPy NCs exhibited significantly lower cell viability under 808-nm NIR laser irradiation compared to the corresponding control experimental samples without NIR laser irradiation. Moreover, simultaneous treatment with 210 μg/mL of CS-PPy NCs and NIR 808-nm laser at 2 W/cm^2^ for 5 min, significantly decreased the cell viability to 25%, while 85% of the viable cells remained alive without irradiation.

In addition, the *in vitro* photothermal effect was observed after the treatment of MDA-MB-231 cells with 210 μg/mL of CS-PPy NCs under different laser power densities (0, 0.5, 1.0, 1.5, and 2.0 W/cm^2^) for 5 min. As shown in Fig. 4f, MDA-MB-231 cells under NIR laser irradiation were obviously less viable with CS-PPy NC treatment than the corresponding control experimental samples without CS-PPy NC treatment. When simultaneously treatment with 210 μg/mL of CS-PPy NCs and NIR 808-nm laser at 2 W/cm^2^ for 5 min, the cell viability was significantly decreased to 25%, while moreover, 85% of cells viability remained alive without irradiation. These results indicated that the combination of CS-PPy NCs and 808-nm NIR laser irradiation could induce localized hyperthermia to ablate cancer cells.

**Figure 5:**
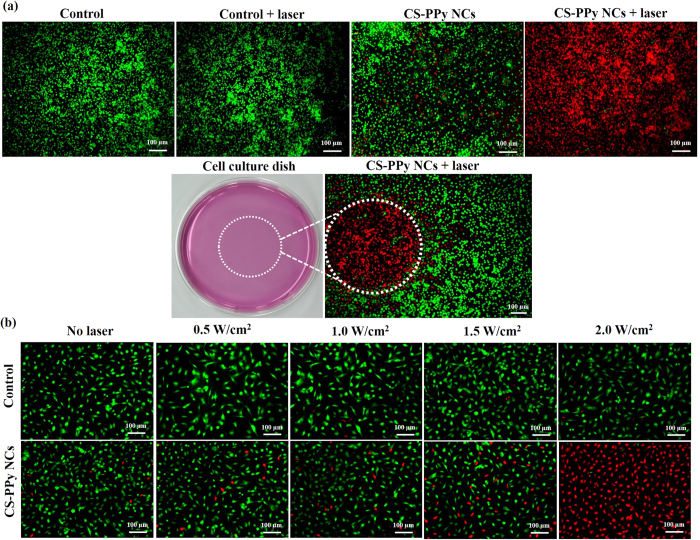
(**a**) Merged fluorescence microscope images of MDA-MB-231 cells treated with different combinations of CS-PPy NCs (210 μg/mL) under 808-nm NIR laser irradiation at 2.0 W/cm^2^ for 5 min (10× magnification): control cells; control cells, 5 min irradiation; CS-PPy NCs of 210 μg/mL only; CS-PPy NCs of 210 μg/mL, 5 min irradiation. The cells are stained by AO (live: green) and PI (dead: red). The culture dish photograph of MDA-MB-231 cells. The white circle indicates the laser spot area. The merged fluorescence image of MDA-MB-231 cells after treatment with 210 μg/mL CS-PPy NCs and NIR laser irradiation in 3.5 cm dish. (**b**) Merged fluorescence microscope images of MDA-MB-231 cells after treatment with or without 210 μg/mL CS-PPy NCs under 808-nm NIR laser irradiation of different laser power densities (0.5 W/cm^2^, 1.0 W/cm^2^, 1.5 W/cm^2^, and 2.0 W/cm^2^) for 5 min (20× magnification). The cells were stained by AO (live: green) and PI (dead: red).

To further examine the *in vitro* photothermal effect of CS-PPy NCs, MDA-MB-231 cells were incubated with 210 μg/mL of CS-PPy NCs and then exposed to an 808-nm NIR laser irradiation at 2 W/cm^2^ for 5 min (Fig. S7). After treatment, cells in all groups were stained with AO and PI to differentiate between the live cells (green fluorescence) and the dead cells (red fluorescence). AO can penetrate live cells and that exhibit green fluorescence. PI can only penetrate the membranes of dead cells that exhibit red fluorescence, indicating an irreversible cellular damage. The merged fluorescence microscope images of all the groups of control cells showed green fluorescence, indicating negligible cell death. The merged fluorescence microscope images of CS-PPy NC- and NIR laser treated cells showed red fluorescence, suggesting that almost all cells were dead, which indicated that CS-PPy NCs could mediate the photothermal destruction of MDA-MB-231 (Fig. 5a). In addition, an *in vitro* photothermal effect was observed after the treatment of the MDA-MB-231 cells with 210 μg/mL of CS-PPy NCs under different laser power densities (0, 0.5, 1.0, 1.5, and 2.0 W/cm^2^) for 5 min (Fig. 5b). Fluorescence imaging of AO and PI stained cells after photothermal ablation also proved the enhanced photothermal effect caused by CS-PPy NCs. Almost all cancer cells were destructed after being incubated with CS-PPy NCs and exposed to the 808-nm NIR laser irradiation at the power density of 2.0 W/cm^2^.

### Cell apoptosis Assay

The cytotoxic effect of photothermal treatment of MDA-MB-231 cells was further quantified by flow cytometry (Fig. S8a). The MDA-MB-231 cells were treated with CS-PPy NCs for 4 h with or without 808-nm NIR laser irradiation and then labeled with Annexin V and PI. The Annexin V and PI cells were defined as late apoptotic or necrotic cells. The results exhibited that CS-PPy NCs and NIR laser irradiation more significantly induced cell late apoptosis or necrosis (82.30%) compared to those treated by laser (1.46%) or CS-PPy NCs (12.20%) (Fig. S8b). The results suggested that the cytotoxic effect of CS-PPy NCs on MDA-MB-231 cells could be boosted by laser irradiation.

### *In vivo* photothermal ablation of cancer cells

**Figure 6:**
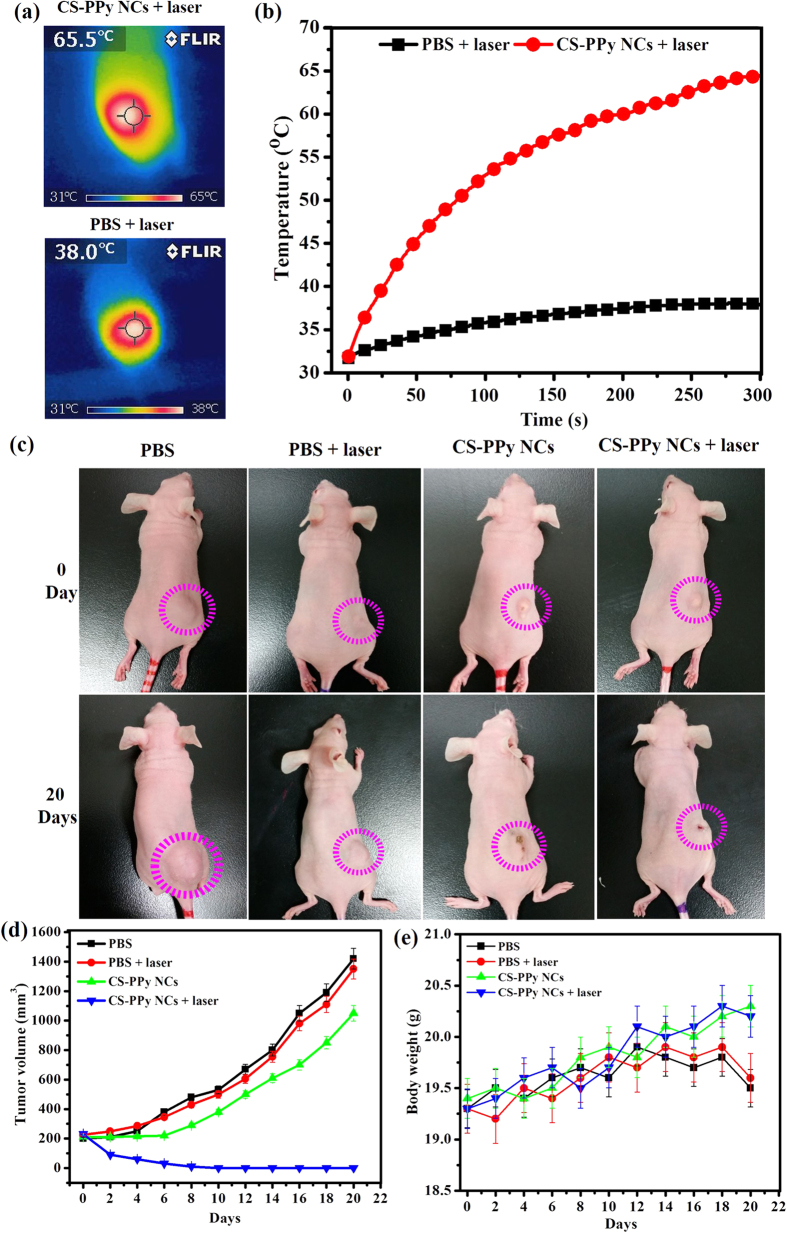
(**a**) NIR thermographic images of tumor-bearing mice with intratumoral injection of PBS and CS-PPy NCs with 808-nm NIR laser irradiation at 2.0 W/cm^2^ for 5 min. (**b**) Temperature change of tumor-bearing mice after intratumoral injection of with PBS and CS-PPy NCs with 808-nm NIR laser irradiation at 2.0 W/cm^2^ for 5 min. (**c**) The digital photographs of tumor-bearing mice taken at day 0 before treatment and 20 days after treatment. Error bars correspond to mean ± standard deviation. (**d**) Tumor volume growth curves of different groups of mice after different treatments. Data presented as mean ± standard deviation. (**e**) The body weight after different treatments indicated in 20 days. Data presented as mean ± standard deviation.

We further examined the *in vivo* photothermal effect of CS-PPy NCs. Tumor-bearing mice were intratumorally injected with CS-PPy NCs. When the volume of the tumor reached ~170 mm^3^, the mice were divided into four groups (n = 5 per group): group I, PBS; group II, PBS + 808-nm NIR laser treatment; group III, CS-PPy NCs only; group IV, CS-PPy NCs + 808-nm NIR laser treatment. Subsequently, the tumor-bearing mice from groups II and IV were treated with 808-nm NIR laser irradiation at 2 W/cm^2^ for 5 min. To monitor the *in vivo* photothermal effect of CS-PPy NCs, temperature changes in the tumor area were noted using a NIR thermal camera (Fig. 6a). As shown in Fig. 6b, the temperature of the tumor areas of mice from CS-PPy NCs + 808-nm NIR laser group could rapidly increase to ~ 65 °C within 5 min, which was high enough to ablate tumors *in vivo*. In contrast, tumors with PBS + 808-nm NIR laser group showed the maximum average temperature lower than 38 °C, which indicates that NIR laser itself could not sufficiently damage tumors.

The tumor volumes were measured in each group and plotted as a function of time (Fig. 6d). Tumors in the control groups (group I, group II, and group III) grew quickly. There was no significant difference between the final tumor sizes of these groups. In contrast, group IV exhibited efficient tumor inhibition and complete eradication of tumors after 20 days photothermal therapy, leaving black scars at the initial tumor sites (Fig. 6c). We also recorded the body weight of the mice through all treatments. In our experiments, no obvious weight loss was observed (Fig. 6e), indicating that the CS-PPy NCs mediated photothermal therapy did not induce any significant systemic toxicity in treated mice. All mice were sacrificed, and tumor tissues were dissected and weighed after 20 days. The tumor photograph for each group is shown in Fig. S9a. The mean tumor weight in the group treated with CS-PPy NCs + 808-nm NIR laser was the lightest among all groups (Fig. S9b). These results showed that PTT based on CS-PPy NCs could cause complete tumor destruction without regrowth during the observation period.

Histological analysis was performed using standard histological techniques with hematoxylin and eosin (H&E) staining to assess the toxicity of CS-PPy NCs on the major organs. On the 25^th^ day of photothermal treatment, the major organs of treated mice were collected for subsequent histological analysis. As shown in Fig. S10, there were no obvious histological changes in the CS-PPy NCs treated mice, which showed no noticeable toxic effect of CS-PPy NCs on the major organs of mice. However, the tumor tissues had disappeared after CS-PPy NCs injection and 808-nm NIR laser irradiation. Cells treated with CS-PPy NCs only exhibited cytotoxicity after NIR laser irradiation, showing several benefits over traditional chemotherapeutic drugs. These results suggested that CS-PPy NCs are promising photothermal agents.

### *In vitro* photoacoustic imaging

**Figure 7:**
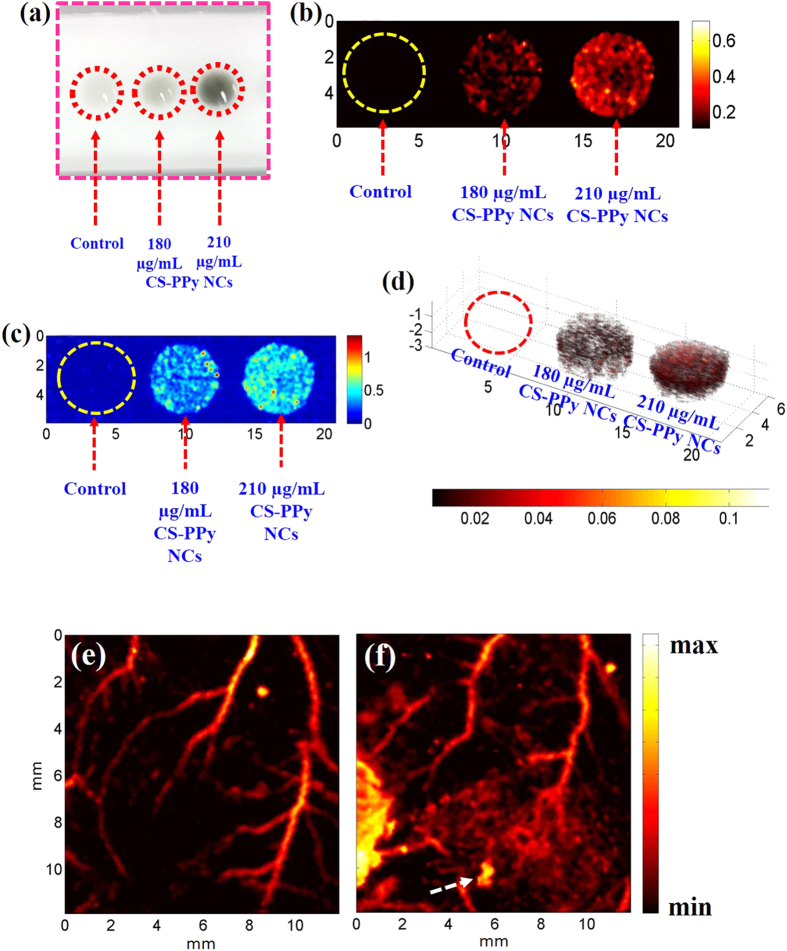
(**a**) Diagram of a tissue mimicking PVA phantom showing a top view structure. (**b** and **c**) Photoacoustic image of control cells and MDA-MB-231 cells treated with two different concentrations of CS-PPy NCs (180 and 210 μg/mL). (**d**) Three-dimensional (3D) photoacoustic image of control cells and MDA-MB-231 cells treated with two different concentrations of CS-PPy NCs (180 and 210 μg/mL). *In vivo* photoacoustic images of tumor areas before (**e**) and after (**f**) injection of CS-PPy NCs in mice tumors. White arrow indicates the intratumoral injection of CS-PPy NCs in mice.

The *in vitro* PAI performance of CS-PPy NCs was evaluated on the PAT system. Figure 7a shows the top of view of the tissue-mimicking PVA phantom including three inclusions of control cells and two different concentrations of CS-PPy NC-treated MDA-MB-231 cells (180 and 210 μg/mL) (Fig. 7b–d). The high-amplitude PA signals were no obvious detect in control cells, suggesting that there were no contrast agents in control cells. The high-amplitude PA signals were detected from the inclusion of high CS-PPy NCs (210 μg/mL) concentration, which indicates the strong efficiency of CS-PPy NC-treated MDA-MB-231 cells to generate PA signals because of the strong NIR absorption inside the cells. These results suggested that CS-PPy NCs can serve as novel contrast agents for image-guided cancer therapy.

### *In vivo* photoacoustic imaging

Polypyrrole nanoparticles have previously been confirmed as contrast agents that can enhance photoacoustic contrast for potential use in non-invasively imaging deep tissues^28^,^36^. PAI can reveal various endogenous absorbers such as hemoglobin, melanin, lipid, and water in biological systems. Exogenous photoacoustic contrast agents will be required to visualize and distinguish tumor tissues from normal tissues by PAI^37^. However, many tumors showed no obvious endogenous photoacoustic contrast owing to their weak optical absorption. *In vivo* therapeutic effects of CS-PPy NCs with strong NIR absorption makes it an excellent candidate for PAI. We tested tumor imaging before intratumoral injection of CS-PPy NCs (Fig. 7e), which suggested that only major blood vessels (*i.e.,* hemoglobin) could be observed at this time. The tumor imaging ability of CS-PPy NCs was examined by a single intratumoral injection of CS-PPy NCs (100 μL of 5 mg/mL) into tumor-bearing mice. The PA image of tumor exhibited greater clarity and a significant increase in the PA signal was detected after injection of CS-PPy NCs (Fig. 7f) in comparison to same region before injection, which indicates a strong efficiency of CS-PPy NCs in generating a PA signal owing to its strong NIR absorption. These results confirmed that CS-PPy NCs could abundantly accumulated in tumor maintaining relatively constant PA signals after a long-time circulation in blood vessels during the entire course of imaging. The distribution of long-circulating CS-PPy NCs in the tumor can afford enhanced and clear PA imaging of tumor of live mice. Hence, the detailed structure of tumor region could be acquired by the CS-PPy NCs enhanced PA imaging, ensuring the precise position of the external NIR laser. In addition, the optimal time-window of photothermal therapy could be chosen base on the intensity of PA signal, as it could reflect the accumulative level of our therapeutic agent in tumor region. These results confirmed that CS-PPy NCs can serve as excellent photothermal agents with enhanced photoacoustic contrast ability, which could provide a promising platform for cancer theranostics^38^.

## Conclusions

Multifunctional biocompatible CS-PPy NCs were successfully fabricated for the photoacoustic imaging-guided photothermal ablation of cancer. CS-PPy NCs showed an excellent photothermal effect because they have strong absorption and scattering in the NIR region. The *in vitro* and *in vivo* photothermal anticancer activity results determine their immense potential in the experiments. The tumor-bearing mice fully recovered after CS-PPy NCs injection as well as NIR 808-nm laser irradiation and the tumor almost disappeared after 20 days. CS-PPy NCs were developed as novel contrast agents for PAI. CS-PPy NCs could act as strong contrast agents to enhance PAI greatly, conducing to accurate locating of cancerous tissue, as well as precise guidance for photothermal therapy. To the best of our knowledge, it is the first report where CS-PPy NCs are used as novel agents for photoacoustic imaging-guided photothermal ablation of cancer. Our work highlights the promising potential of CS-PPy NCs in the photoacoustic imaging-guided photothermal ablation of cancer.

## Materials and Methods

### Chemicals

CS (low molecular weight, ~310 kDa; degree of deacetylation, 90%) was purchased from Kitto Life co., Ltd. (Seoul, South Korea). Pyrrole (98%), 3-(4,5-dimethylthiazol-2-yl)-2,5-diphenyltetrazolium bromide (MTT), acridine orange (AO), propidium iodide (PI), and polyvinyl alcohol (PVA) were purchased from Sigma–Aldrich Co. (St. Louis, MO, USA). Silica was obtained from Min-U-Sil (Min-U-Sil, U.S silica, Pacific, MO, USA). All other chemicals were of analytical grade and they were purchased from Sigma–Aldrich Co. (St. Louis, MO, USA).

### Synthesis of chitosan-polypyrrole nanocomposites

The chitosan-polypyrrole nanocomposites (CS-PPy NCs) were synthesized according to previously reported method^39^. 0.15 g CS was dissolved in 30 mL 0.25% acetic acid solution and 0.90 g FeCl_3_·6H_2_O was added in the chitosan aqueous solution while stirring at room temperature. After the solution completely dissolved, 100 μL of pyrrole was added into the chitosan aqueous solution slowly under 0–5 °C ice water bath conditions. The color of the solution quickly turned in black and it was further stirred for 1 h, indicating the formation of CS-PPy NCs. The formation of CS-PPy NCs was recorded using UV-Vis spectroscopy (Beckman Coulter, Fullerton, CA, USA). Subsequently, the sample was purified using a dialysis tube (2000 MWCO, Sigma–Aldrich Co., St. Louis, MO, USA), via dialysis against distilled water for three days to thoroughly remove iron ions, followed by lyophilization.

### Characterization of CS-PPy NCs

The synthesized CS and CS-PPy NCs were analyzed according to their X-ray diffraction (XRD) patterns. XRD was measured using a Philips X’Pert-MPD PW 3050 diffractometer with Cu Kα (40 KV, 30 mA) in the step-scan mode. The CS and CS-PPy NCs were examined via Fourier transform infrared spectroscopy (FTIR, Perkin Elmer Inc., USA) with frequencies ranging from 4000 to 400 cm^−1^. Particle size and morphology of CS-PPy NCs were studied using field emission transmission electron microscopy (FETEM; JEM-2100F, JEOL, Japan). Particle size and size distribution of CS-PPy NCs were analyzed using dynamic light scattering (DLS) and zeta potential (ZP) measurements were performed using an electrophoretic light scattering spectrophotometer (ELS-8000, OTSUKA Electronics Co. Ltd., Japan) with an ELS FlatBoard cell and a zeta potential analyzer at room temperature. The stability study of CS-PPy NCs was performed at room temperature. Changes in surface plasmon resonance (SPR) dispersion of the CS-PPy NCs were recorded for up to 6 months using UV-Vis spectroscopy.

### Photothermal heating performance

Different concentrations of CS-PPy NCs (10, 30, 60, 90, 120, 150, 180, and 210 μg/mL) dispersed in distilled water (1 mL) were irradiated using a 808-nm fiber-coupled laser (Changchun New Industries Optoelectronics Technology, Changchun, China) at different power densities (0.5, 1.0, 1.5, and 2.0 W/cm^2^) for 5 min. Sample temperature was observed and confirmed every 1 s by a digital thermometer with a thermocouple probe. As control, distilled water was irradiated using an 808-nm NIR laser. The heating curve was determined by plotting the measured temperatures. The CS-PPy NCs (210 μg/mL) solutions were irradiated using an 808-nm NIR laser at 2 W/cm^2^ for 5 min (LASER ON), followed by natural cooling at room temperature for 15 min without 808-nm NIR laser irradiation (LASER OFF). This cycle was repeated five times and the UV-vis-NIR spectra of the irradiated samples were obtained for characterizing the photostability of their absorption properties^28^.

### Cell culture

A human breast cancer cell line (MDA-MB-231) and human embryonic kidney cell line (HEK 293) were cultured in standard culture media recommended by the Korean Cell Line Bank. Cells were incubated at 37 °C in a humidified atmosphere containing 5% CO_2_.

### *In vitro* cytotoxicity assay

Toxicity of the CS-PPy NCs was tested on MDA-MB-231 cells. The MDA-MB-231 cells were seeded into 96-well plates at a density of 1 × 10^4^ cells/well and they were incubated for 24 h at 37 °C in 5% CO_2_ atmosphere. After incubation, the cells were treated with varying concentrations of the CS-PPy NCs (50, 100, 150, 200, 250, 300, 350, 400, 450, and 500 μg/mL) for 24 h and 48 h to measure cytotoxicity using the MTT assay.

### Biocompatibility study

HEK 293 cells were seeded into 96-well plates at a density of 1 × 10^4^ cells/well and they were incubated for 24 h at 37 °C in a 5% CO_2_ atmosphere. The medium was replaced with fresh complete media containing CS-PPy NCs concentrations ranging from 30 to 510 μg/mL. The plate was further incubated for 24 h and 48 h followed by analysis of cell viability using the MTT assay.

### *In vitro* photothermal ablation of cancer cells

To evaluate the photothermal effect of CS-PPy NCs on breast cancer cells, MDA-MB-231 cells were seeded into 96-well plates at a density of 1 × 10^4^ cells/well and they were incubated for 24 h. After incubation, the culture medium was removed and cells were treated with different concentrations of CS-PPy NCs (10, 30, 60, 90, 120, 150, 180, and 210 μg/mL) at 37 °C for 4 h. Cells were then treated with or without 808-nm NIR laser irradiation at 2 W/cm^2^ for 5 min. After irradiation treatment, the cells were then incubated at 37 °C for 2 h and cell viability was measured using the MTT assay.

MDA-MB-231 cells were seeded into 96-well plates at a density of 1 × 10^4^ cells/well and they were incubated for 24 h at 37 °C. After incubation, the culture medium was removed, and cells were randomly divided into four groups: group I, blank control cells; group II, cells +808-nm NIR laser treatment; group III, CS-PPy NCs (210 μg/mL) only; group IV, CS-PPy NCs (210 μg/mL) +808-nm NIR laser treatment. The cells were treated with CS-PPy NCs (210 μg/mL) at 37 °C for 4 h. After incubation, the cells in groups II and IV were exposed to an 808-nm laser at different power densities (0.5, 1.0, 1.5, and 2.0 W/cm^2^) for 5 min. After irradiation treatment, cells were then incubated at 37 °C for 2 h and cell viability was determined using the MTT assay.

MDA-MB-231 cells were seeded into 6-well plates at a density of 2 × 10^5^ cells/well and they were incubated for 24 h at 37 °C. After incubation, the cells were treated with CS-PPy NCs (210 μg/mL) at 37 °C for 4 h. Subsequently, the cells of groups II and IV were treated with NIR laser irradiation at 2 W/cm^2^ for 5 min. After incubation for additional 2 h, the cells in all groups were stained with acridine orange (AO) and propidium iodide (PI) for the assessment of photothermal effect and a Leica DMI300B fluorescence microscope (Leica Microsystems, Wetzlar, Germany) was used to obtain fluorescence images of the samples.

### Cell apoptosis assay

Cytotoxicity of PTT was determined by quantification of apoptotic or necrotic cancer cells. MDA-MB-231 cells were seeded at a density of 4 × 10^5^ cells/well in 24-well plates for 24 h at 37 °C and they were then treated with or without CS-PPy NCs (210 μg/mL) for 4 h. After washing, the cells were treated with or without the 808-nm laser at 2 W/cm^2^ for 5 min. Subsequently, cells were cultured for additional 2 h and they were then harvested. Fluorescein isothiocyanate Annexin V Apoptosis Detection Kit (BD Biosciences, USA) was used to detect and quantify apoptosis using a flow cytometer (BD FACSVerse, NJ, USA). Annexin V-positive and PI-negative cells were recorded as apoptotic. Double-stained cells were considered to be apoptotic or necrotic.

### Animals and tumor model

Female BALB/c nude mice weighing 18–21 g were obtained from Orient Bio Inc. (Gyeonggi-Do, Korea), and they were housed in stainless steel cages under standard conditions (20 ± 2 °C room temperature, 60 ± 10% relative humidity) with a 12 h light/dark cycle. All described procedures were reviewed and approved by Pukyong National University animal care and use committee, and performed in accordance with the guiding principles for the care and use of laboratory animals. MDA-MB-231 cells (4 × 10^6^) were subcutaneously injected into the flank region of the mice.

### *In vivo* photothermal ablation of cancer cells

Tumor-bearing mice were intratumorally injected with 100 μL of 5 mg/mL CS-PPy NCs when the tumor volume reached ~170 mm^3^. Mice were divided into four groups (n = 5): group I, PBS; group II, PBS + 808-nm NIR laser treatment; group III, CS-PPy NCs only; group IV, CS-PPy NCs + 808-nm NIR laser treatment. Subsequently, tumor-bearing mice in groups II and IV were treated with NIR laser irradiation at 2 W/cm^2^ for 5 min. Before irradiation, a thermometer probe was inserted into the tumor center. Tumor temperature was recorded every 1 s during irradiation. Temperature change in the tumor region under laser irradiation was recorded for all groups using a FLIR i5 infrared camera (Flir Systems Inc., Portland, USA). Tumor growth and mouse weight were measured in the following days. After PTT, tumor dimensions were measured every two days using a caliper and tumor volume was calculated according to the formula (1):


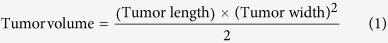


After finalization of the experiments, mice were sacrificed, and tumors and major organs were dissected and weighed to evaluate the therapeutic efficiency of photothermal ablation in different groups.

### Histological analysis

For histology, major organs such as the heart, liver, spleen, lung, and kidney of mice were harvested and fixed in 10% neutral buffered formalin, processed routinely into paraffin, cut into 4 μm thick sections, and stained with hematoxylin and eosin (H&E). The stained samples were visualized under the light microscope.

### *In vitro* photoacoustic imaging

*In vitro* photoacoustic imaging was described according to the literature^40^. A phantom that closely mimics human tissue was prepared for photoacoustic imaging of CS-PPy NCs-treated MDA-MB-231 cells (180 and 210 μg/mL). PVA phantoms were fabricated using 8% PVA, 0.4% silica, and 100 mL distilled water. Control cells and CS-PPy NCs-treated MDA-MB-231 cells were mixed with 10% gelatin and used as three inclusion (50 μL each/well) in the tissue-mimicking PVA phantom.

### *In vivo* photoacoustic imaging

The photoacoustic tomography (PAT) system was described by Bui *et al*.^41^. The PAT system is based upon an optical parametric oscillator (OPO) laser (Surelite OPO Plus, Continuum, CA, USA) pumped by a Nd:YAG laser (Surelite III, Continuum, CA, USA) with a pulse width of 5 ns and a repetition rate of 10 Hz. The OPO operated at 808-nm was used to irradiate the samples with an incident laser fluence below 9 mJ/cm^2^ for generating photoacoustic (PA) signals. The PA signals were captured by a 10 MHz focused transducer (Olympus NDT, Waltham, MA, USA) and they were then digitized and stored by a data acquisition system in synchronization with a laser system. Finally, the detected PA signals were converted into PA images via Hilbert transformation. The scanning step sizes for the x and y directions were 100 and 100 μm, respectively. For a 12 × 12-nm field of view, the acquisition time was 44 min. Photoacoustic information of tumor areas before and after intratumoral injection of CS-PPy NCs in mice was acquired under 808-nm excitation.

### Statistical Analysis

Data were expressed as mean ± standard deviation (SD). All statistical analyses were performed using the SPSS software version 14.0 (SPSS Inc., Chicago, IL, USA) and significant differences between the groups were determined using one-way ANOVA analysis.

## Additional Information

**How to cite this article:** Manivasagan, P. *et al*. Multifunctional biocompatible chitosan-polypyrrole nanocomposites as novel agents for photoacoustic imaging-guided photothermal ablation of cancer. *Sci. Rep.* **7**, 43593; doi: 10.1038/srep43593 (2017).

**Publisher's note:** Springer Nature remains neutral with regard to jurisdictional claims in published maps and institutional affiliations.

## Supplementary Material

Supplementary Figures
