# Supplementary material for: Multifunctional biocompatible chitosan-polypyrrole nanocomposites as novel agents for photoacoustic imaging-guided photothermal ablation of cancer
Source: Sci Rep. 2017 Mar 2;7:43593. doi: 10.1038/srep43593 (PMC5333628; doi:10.1038/srep43593)
Supplement: Supplementary Figures [file srep43593-s1.pdf]

Multifunctional biocompatible chitosan-polypyrrole nanocomposites as novel agents for photoacoustic imaging-guided photothermal ablation of cancer

Panchanathan Manivasagan[1], Nhat Quang Bui[2], Subramaniyan Bharathiraja[1], Madhappan Santha Moorthy[1], Yun-Ok Oh[1], Kyeongeun Song[2], Seo Hansu[2], Min Yoon[3] & Junghwan Oh[1.2,*]

[1]Marine-Integrated Bionics Research Center, Pukyong National University, Busan 48513, Republic of Korea.

[2]Department of Biomedical Engineering and Center for Marine-Integrated Biotechnology (BK21 Plus), Pukyong National University, Busan 48513, Republic of Korea.

[3]Department of statistics, College of Natural of Sciences, Pukyong National University, Busan 48513, Republic of Korea.

*** Corresponding author**

Prof. Junghwan Oh[1.2,*]

[1]Marine-Integrated Bionics Research Center, Pukyong National University, Busan 48513, Republic of Korea.

[2]Department of Biomedical Engineering and Center for Marine-Integrated Biotechnology (BK21 Plus), Pukyong National University, Busan 48513, Republic of Korea.

Email: jungoh@pknu.ac.kr (J. Oh). Tel: +82-51-629-5771, Fax: +82-51-629-5779.

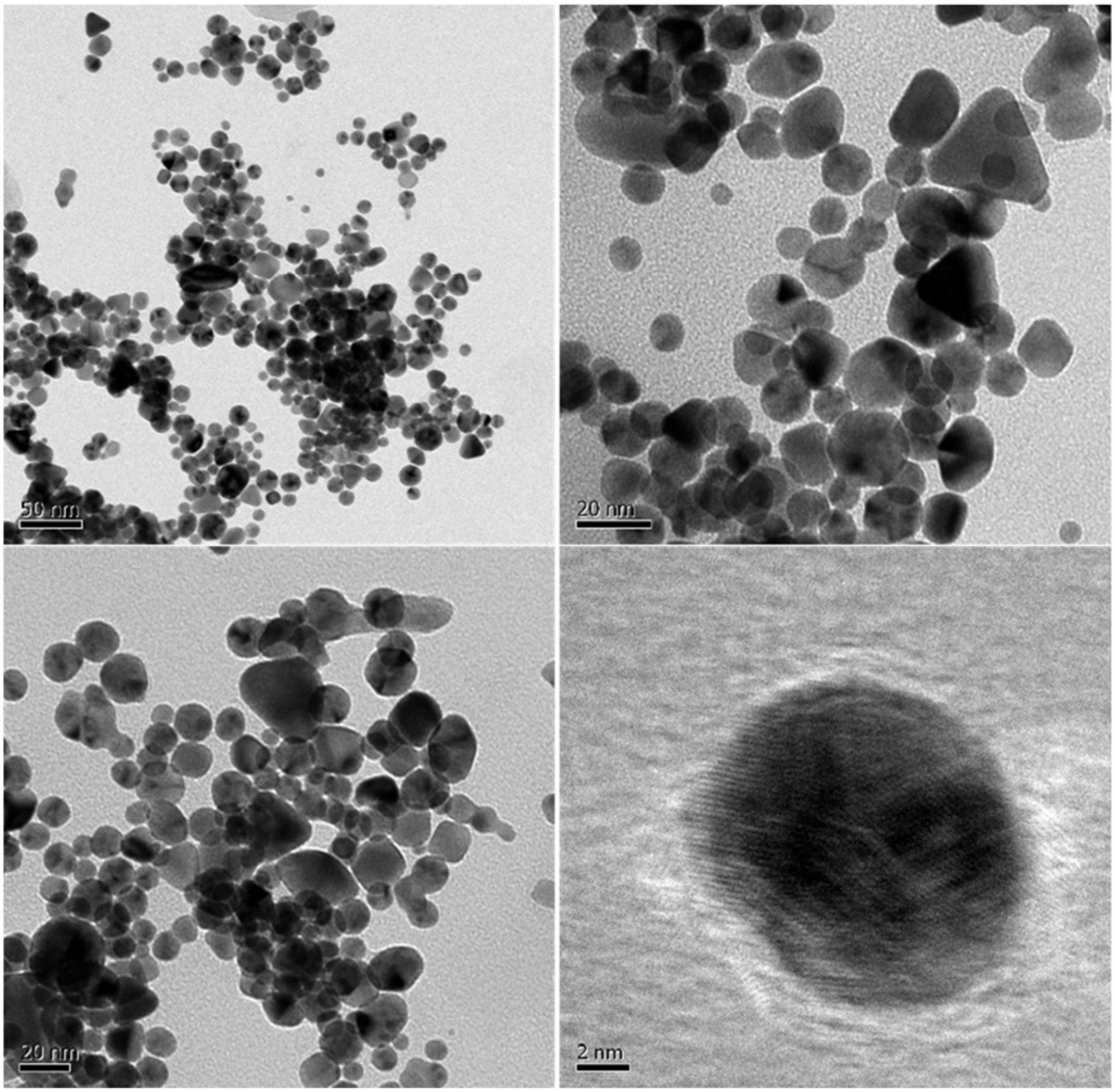

Figure S1. FETEM image of chitosan-polypyrrole nanocomposites (CS-PPy NCs).

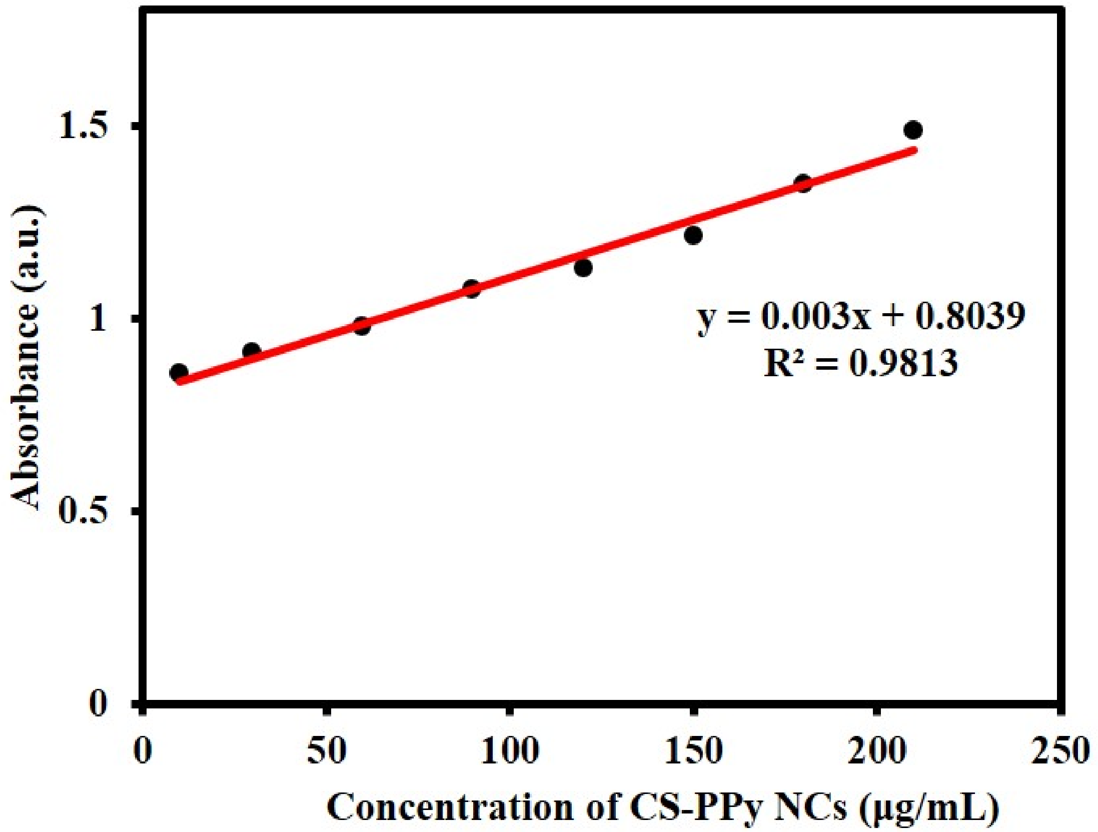

Figure S2. A linear relationship for the absorbance at 808-nm wavelength as a function of the concentration.

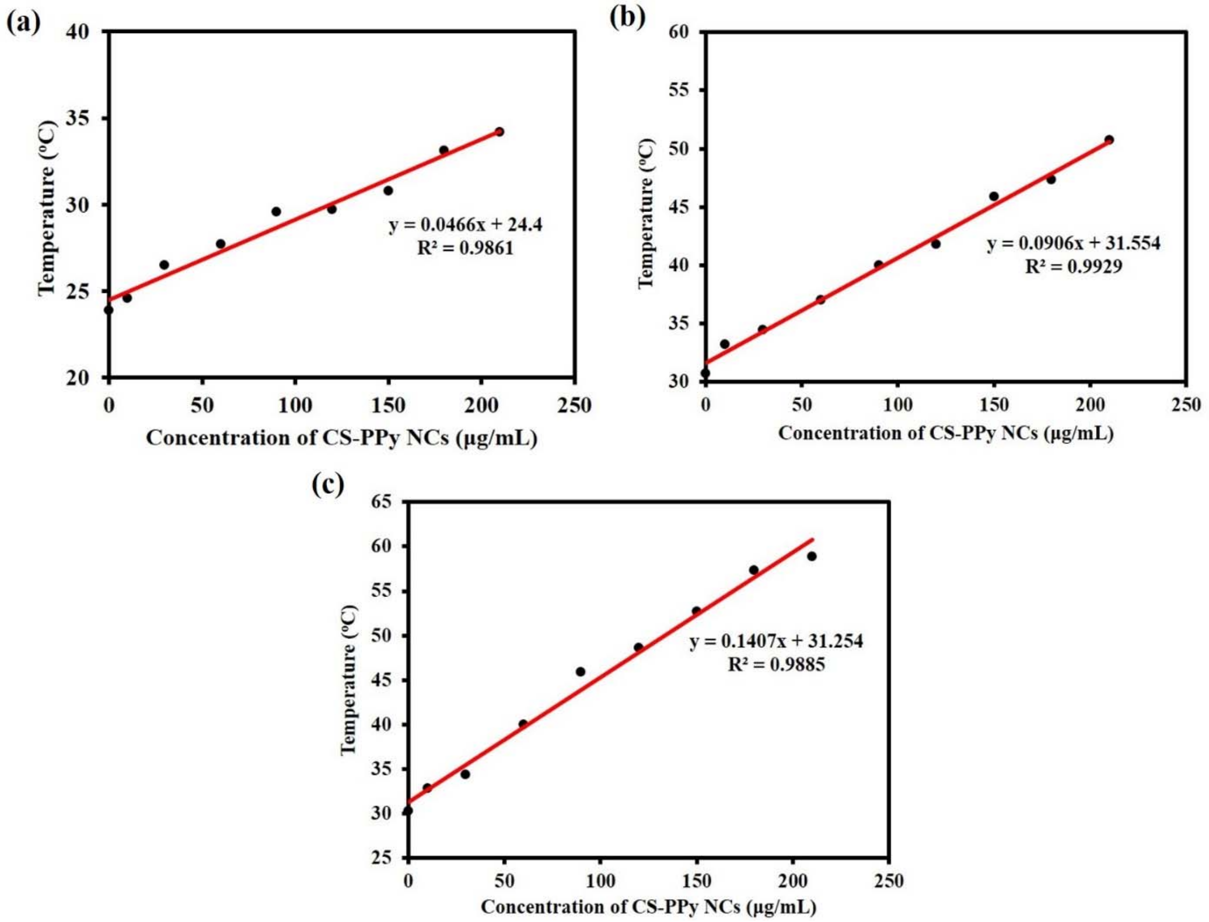

Figure S3. Linear fitting of temperature elevation at different CS-PPy NCs concentrations as a function of irradiation time by 808 nm NIR laser at 1.0 (a), 1.5 (b), and 2.0 W/cm^2 (c).

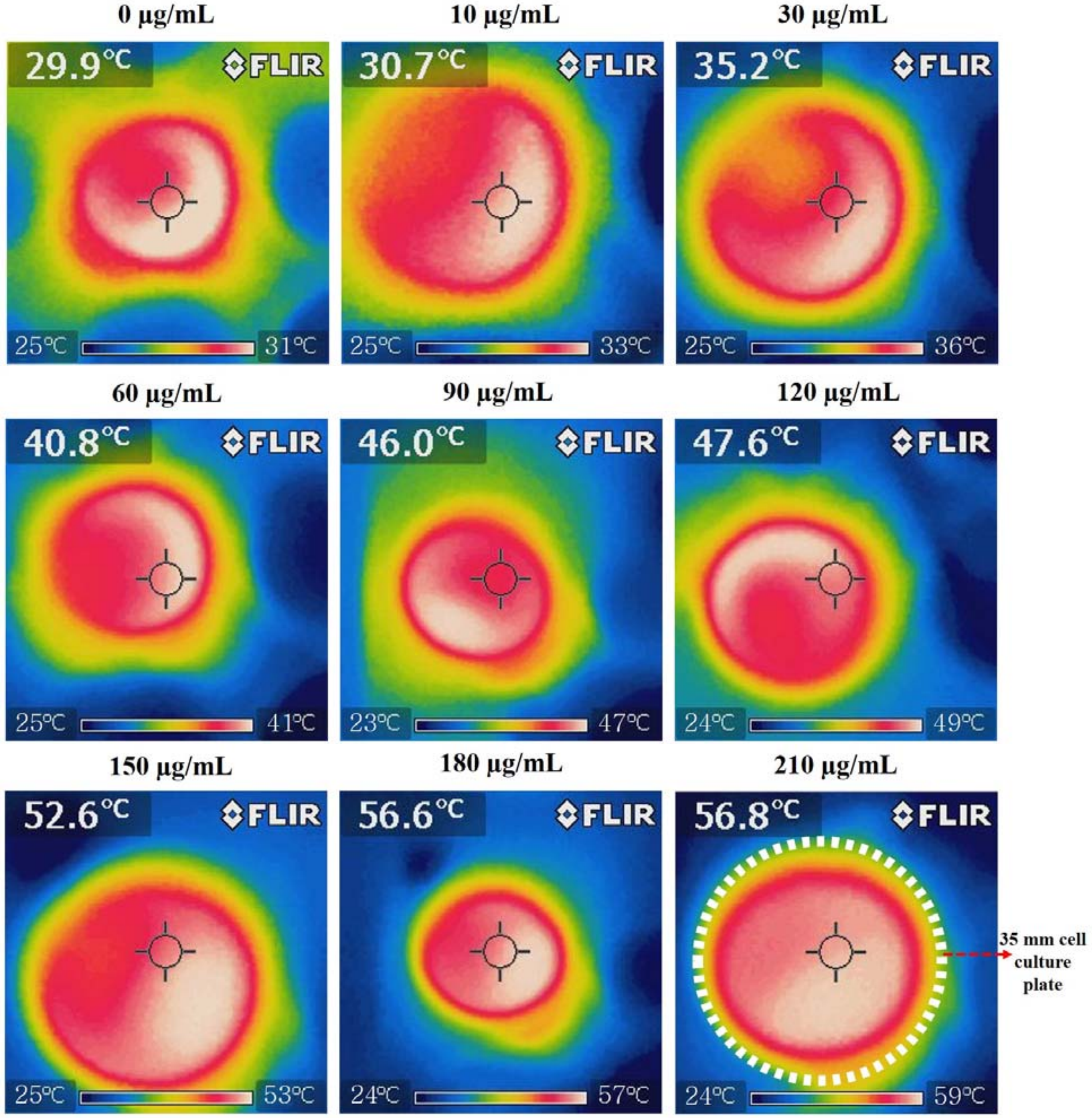

Figure S4. NIR thermographic images of different CS-PPy NC concentrations (0, 10, 30, 60, 90, 120, 150, 180, and 210 μg/mL) aqueous solution under exposure to an 808-nm NIR laser at 2.0 W/cm^2 for 5 min.

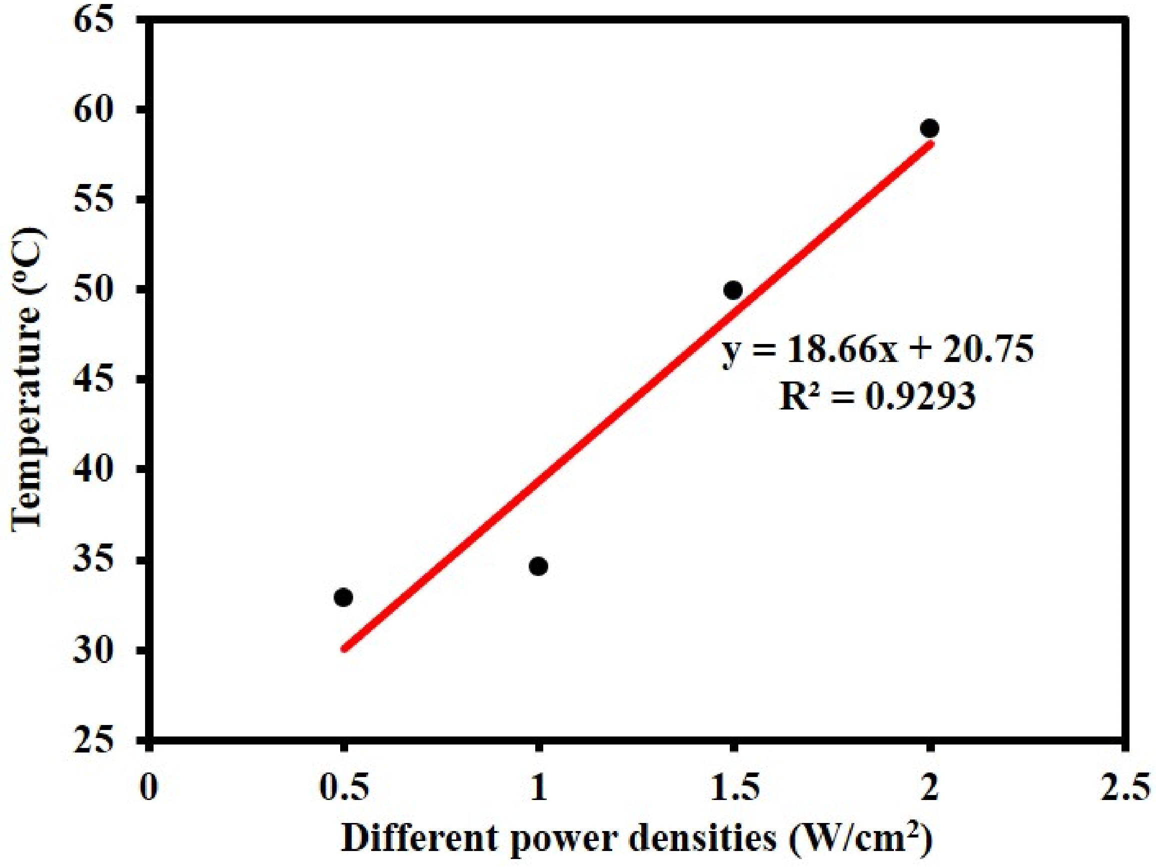

Figure S5. Linear fitting of temperature elevation of 210 μg/mL CS-PPy NCs solution under 808-nm laser irradiation at different power densities (0.5 W/cm^2, 1.0 W/cm^2, 1.5 W/cm^2, and 2.0 W/cm^2) for 5 min.

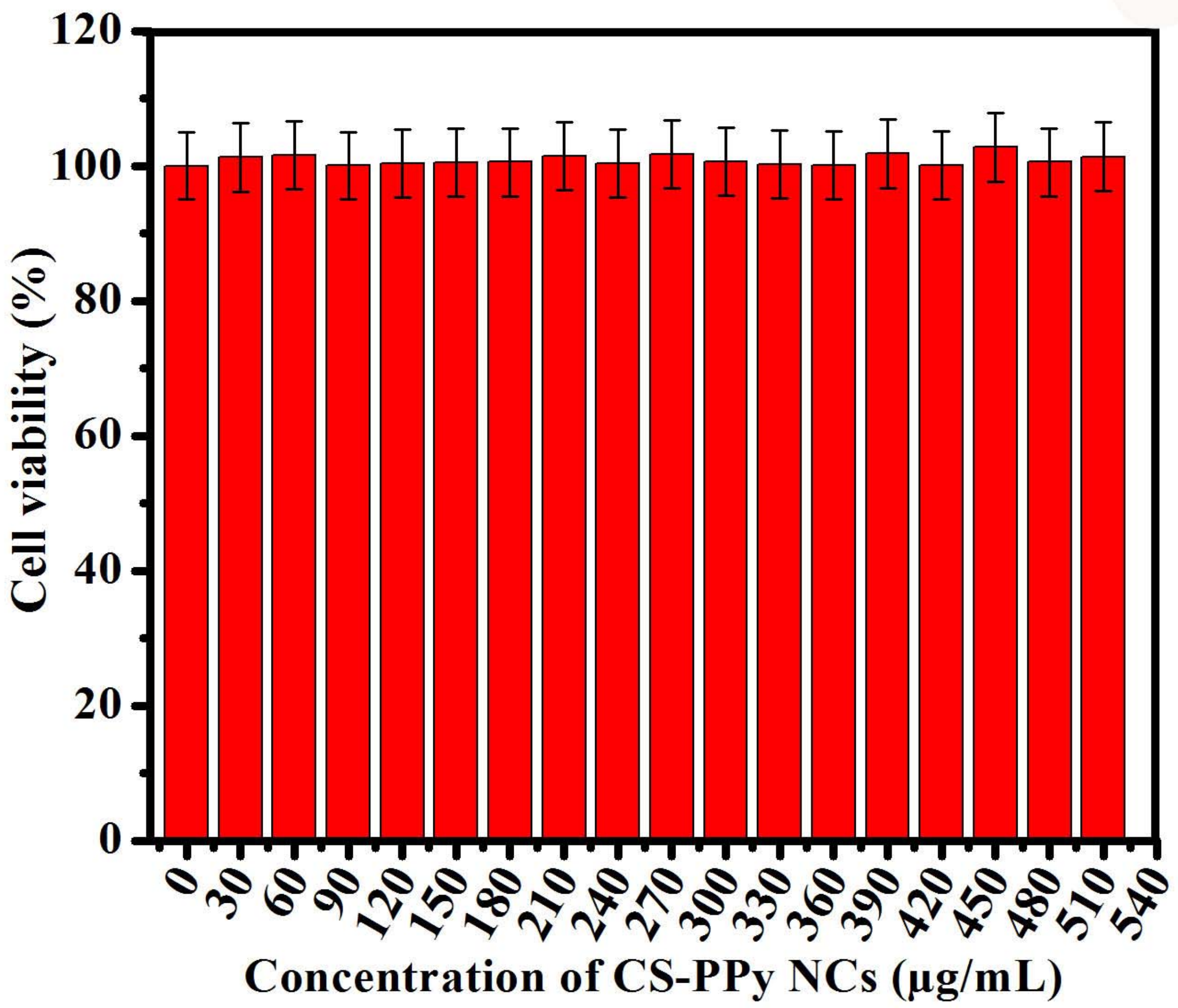

Figure S6. Biocompatibility of CS-PPy NCs against HEK 293 cells for 48 h. Data presented as mean ± standard deviation (n = 3).

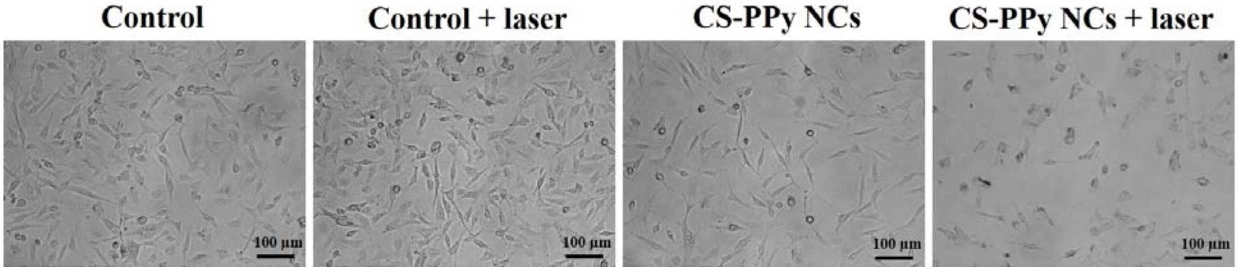

Figure S7. Morphological changes in MDA-MB-231 cells treated with different combinations of CS-PPy NCs (210 μg/mL) under 808-nm NIR laser irradiation at 2.0 W/cm^2 for 5 min (20 × magnification): control cells; control cells, 5 min irradiation; CS-PPy NCs of 210 μg/mL only; CS-PPy NCs of 210 μg/mL + 5 min irradiation.

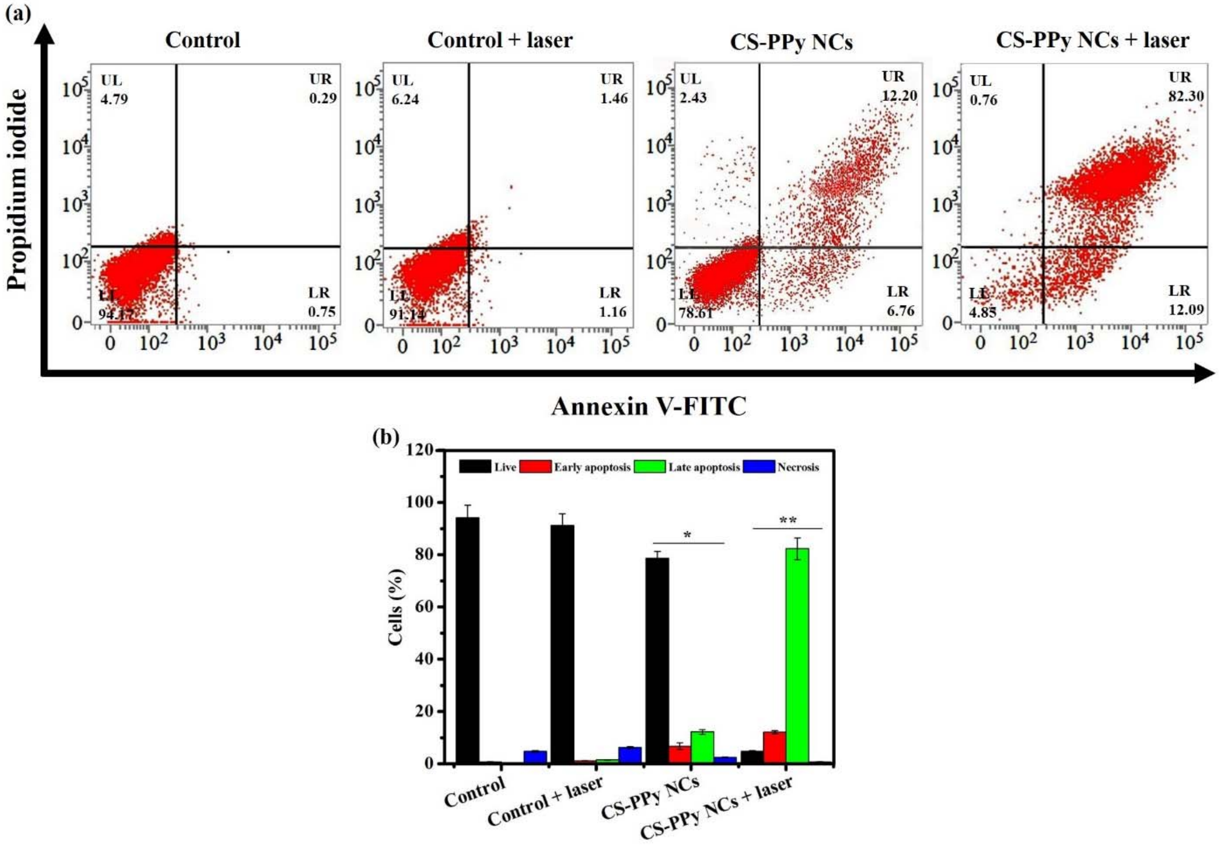

Figure S8. (a) Flow cytometry analysis of MDA-MA-231 cells treated by different combinations of CS-PPy NCs (210 μg/mL) under 808-nm NIR laser irradiation at 2.0 W/cm^2 for 5 min. (b) Quantified analysis of apoptotic and necrotic cell percentage according to double staining by Annexin V and PI (* significant *p* < 0.05; ** highly significant *p* < 0.01).

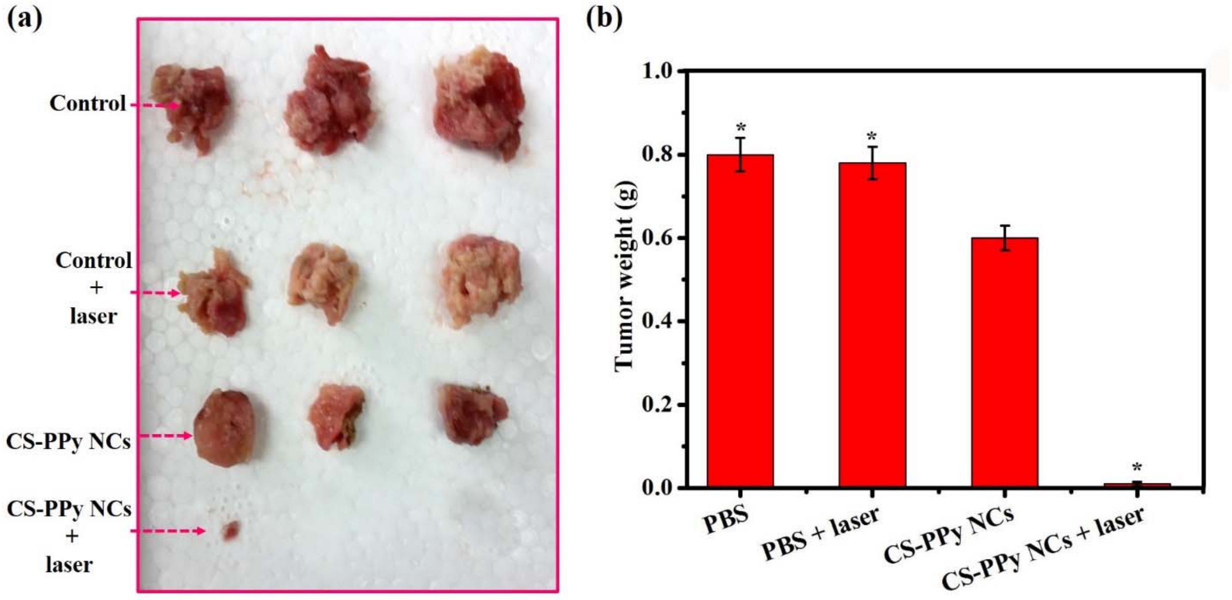

Figure S9. (a) The digital photographs of tumors collected from different groups of mice at the end of treatment. (b) Average weights of tumors collected from the mice at the end of photothermal therapy (* significant $p < 0.05$).

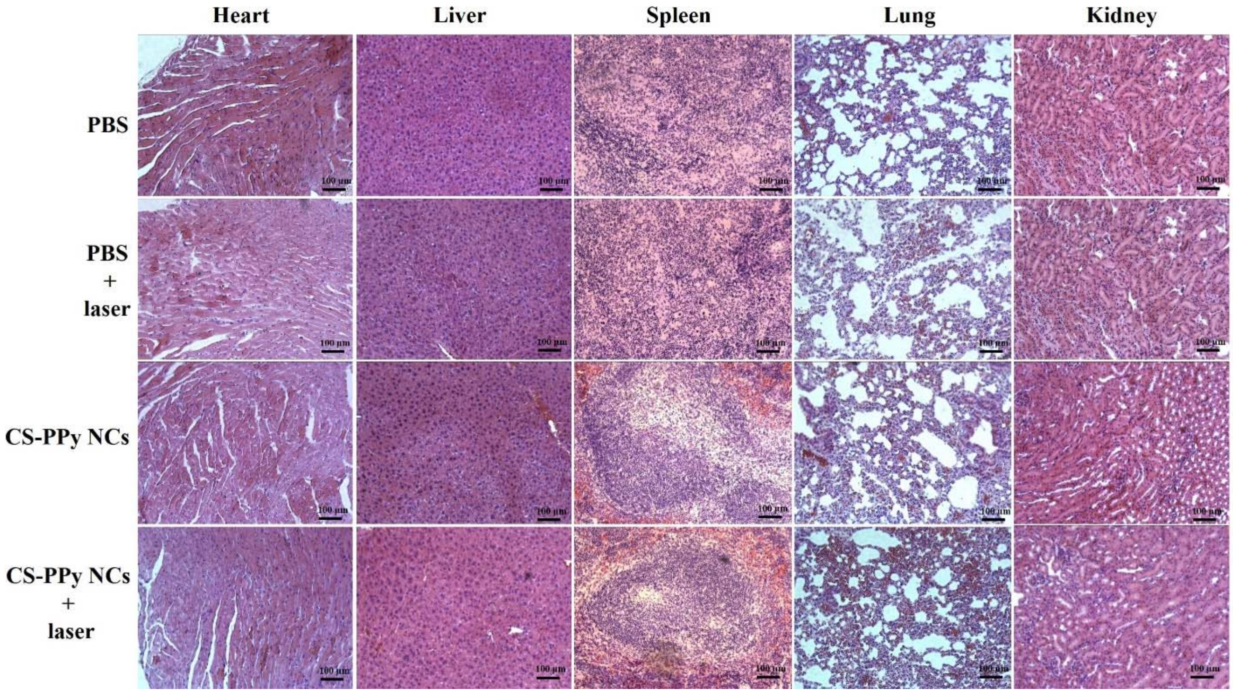

Figure S10. Pathological analysis: hematoxylin and eosin (H&E) stained sections of major organs collected from different treatment groups on the 20th day.
